# Orthodontic Management of Vertical Maxillary Excess With Occlusal Cant and Molar Protraction in a Young Adult Female: A Case Report

**DOI:** 10.7759/cureus.103590

**Published:** 2026-02-14

**Authors:** Harsh A Mishra, Shashank Gaikwad, Parag Gangurde, Hitesh R Sawant, Sakshi Khedekar

**Affiliations:** 1 Department of Orthodontics and Dentofacial Orthopedics, Bharati Vidyapeeth (Deemed to be University) Dental College and Hospital, Navi Mumbai, IND

**Keywords:** class ii malocclusion, gummy smile, infrazygomatic crest implants, maxillary intrusion, molar protraction, mutilated dentition, occlusal cant, orthodontic biomechanics, temporary anchorage devices, vertical maxillary excess

## Abstract

Vertical maxillary excess (VME) often presents with excessive gingival display, deep bite, and occlusal plane discrepancies that significantly impact smile esthetics. The management of VME in adult patients with compromised dentition poses unique challenges requiring comprehensive treatment planning and precise biomechanical execution. This case report describes the diagnosis and orthodontic management of a 19-year-old female presenting with a VME-induced gummy smile, occlusal cant, increased overjet, and a decayed mandibular molar requiring extraction. The patient exhibited a skeletal Class II maxillomandibular relationship with end-on canine and molar relations bilaterally, excessive gingival display exceeding 4 mm, and a mutilated mandibular first molar (tooth 36) with a mesioangularly erupting third molar (tooth 38). The patient was diagnosed with VME, excessive gingival display, occlusal cant on the right side, a deep bite of 4 mm, increased overjet of 5 mm, Class II molar and canine relations on the left side, a non-restorable mandibular molar requiring extraction, and midline discrepancy. Following the extraction of the compromised mandibular first molar, space was successfully managed through orthodontic molar protraction. The mandibular second molar (tooth 37) was protracted into the extraction space of tooth 36, achieving a bilateral Class I molar relationship. Bilateral premolar extractions in the upper arch created space for simultaneous whole arch intrusion assisted by temporary anchorage devices in the maxillary anterior region and infrazygomatic crest implants in the maxillary posterior region. Comprehensive fixed mechanotherapy corrected vertical and sagittal discrepancies, re-established symmetry, improved smile esthetics, and resolved the occlusal cant. This report highlights the importance of carefully planned biomechanics and vertical smile management in addressing esthetic imbalances without surgical intervention.

## Introduction

Adult orthodontics encompasses a wide array of clinical challenges due to the presence of mutilated teeth, compromised anchorage preparation, and skeletal discrepancies that significantly affect treatment planning and execution. Vertical maxillary excess (VME) is a common etiology behind excessive gingival display, deep bite, and unesthetic smile lines [1-3]. Increased vertical display during smiling is perceived as non-esthetic and represents a primary concern for many patients seeking orthodontic treatment. Patients often report dissatisfaction related more to their smile proportion and gingival display than to crowding or tooth irregularity alone.

Managing VME can be challenging, typically requiring differential intrusion mechanics, occlusal plane correction, or multidisciplinary intervention involving orthognathic surgery [4-6]. The advent of temporary anchorage devices (TADs) and infrazygomatic crest implants (IZCs) has revolutionized orthodontic treatment by providing absolute anchorage, thereby enabling controlled intrusion mechanics without taxing dental units [7-9]. These skeletal anchorage systems have expanded the treatment envelope for non-surgical management of vertical discrepancies.

Space management due to mutilated teeth often complicates treatment mechanics, particularly when asymmetric extractions are required in treatment planning. The decision between prosthetic replacement and orthodontic space closure through molar protraction represents a critical treatment planning consideration. Molar protraction is a biologically favorable method that preserves bone, eliminates prosthetic needs, and maintains periodontal integrity [10-12]. However, it requires careful biomechanical planning to prevent undesirable tipping and ensure proper root parallelism.

Occlusal cant correction adds another layer of complexity to treatment planning, requiring asymmetric mechanics and controlled vertical elastics to achieve facial symmetry [13]. The combination of VME, occlusal cant, compromised dentition, and skeletal discrepancies necessitates thorough planning and meticulous execution of planned treatment mechanics to ensure successful attainment of treatment objectives.

This case report presents a detailed orthodontic management plan for a young adult female patient with VME, gummy smile, occlusal cant, and a compromised mandibular molar. The treatment approach used non-surgical vertical control through anterior intrusion, occlusal cant correction, and molar protraction into an extraction space as an alternative to prosthetic restoration or implant placement. This report highlights the importance of carefully planned biomechanics and vertical smile management in addressing complex esthetic and functional imbalances in adult orthodontic patients.

## Case presentation

Patient information

A 19-year-old female patient presented to the orthodontic clinic with the chief complaint of excessive gum display while smiling and irregular teeth. The patient expressed significant concern regarding her smile esthetics and desired improvement in her facial appearance. Medical history was non-contributory, with no systemic diseases or contraindications to orthodontic treatment. The patient had good oral hygiene and was motivated for comprehensive orthodontic treatment.

Clinical examination

Extraoral Findings

**Figure 1:**
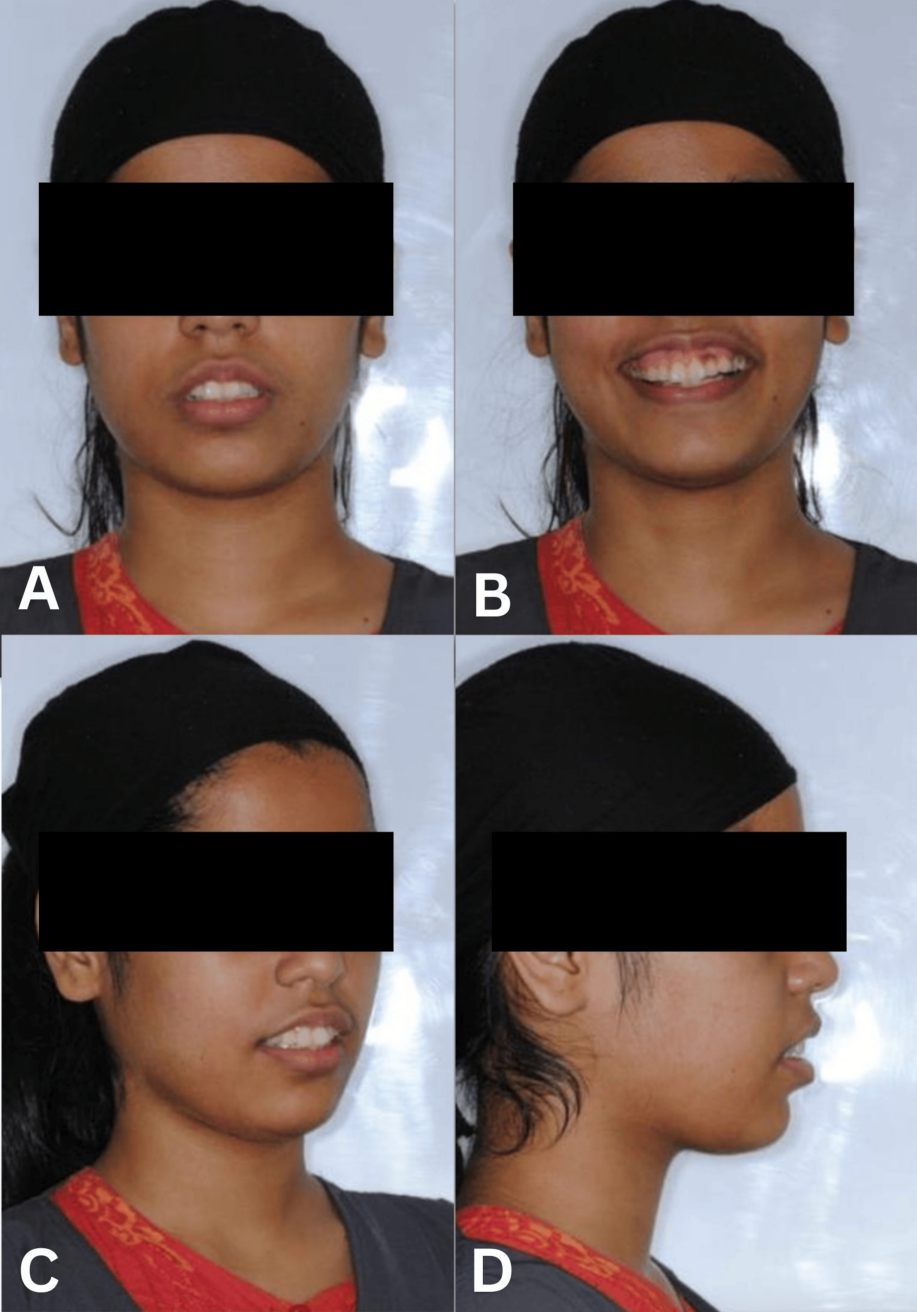
Pre-treatment extraoral photographs (frontal and profile views) demonstrating a convex facial profile, increased lower anterior facial height, excessive gingival display (>4 mm), flat smile arc, and occlusal cant dipping on the right side.

Facial examination revealed a convex facial profile with increased lower anterior facial height. The patient exhibited excessive gingival display exceeding 4 mm during smiling, with a flat smile arc that contributed to the unesthetic appearance. Incisor display at rest was reduced, while smiling produced exaggerated gingival exposure. A notable occlusal cant was present, dipping on the right side, which contributed to facial asymmetry. The patient’s facial proportions showed VME with disproportionate lower facial height (Figure 1).

Intraoral Findings

**Figure 2:**
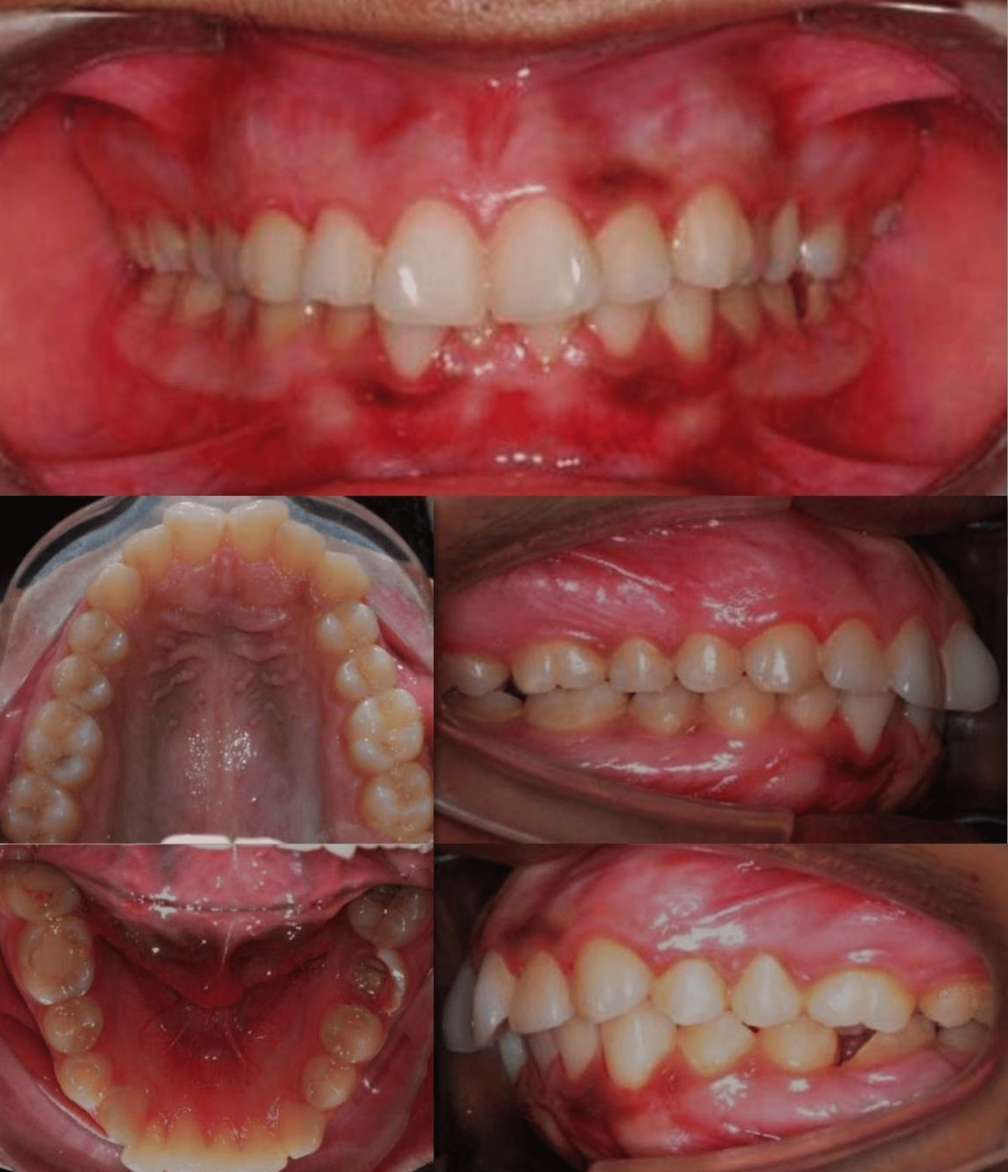
Pre-treatment intraoral photographs (intraoral views) showing a Class I molar relationship on the right side and Class II on the left side, increased overjet (5 mm) and overbite (4 mm), non-coincidence midlines, occlusal cant, and decayed mandibular first molar (tooth 36).

Intraoral examination revealed a Class I molar relationship on the right side and a Class II molar relationship on the left side. The canine relationship was Class I on the right side and Class II on the left side. The patient presented with an overjet of 5 mm and an overbite of 4 mm, with non-coincident dental midlines. The occlusal plane demonstrated a cant toward the right side. Gingival display exceeded 4 mm during smile, with a U-shaped arch form that was asymmetrical. A severely decayed mandibular first molar (tooth 36) was identified, which was deemed non-restorable and required extraction. The mandibular third molar (tooth 38) was mesioangularly erupting (Figure 2).

Diagnostic Records

Comprehensive diagnostic records were obtained before treatment initiation, including extraoral photographs (frontal, profile, and smiling views), intraoral photographs (frontal, lateral, and occlusal views), a lateral cephalometric radiograph, orthopantomogram (OPG), intraoral periapical radiographs (IOPAs), and study models.

Radiographic Findings

**Figure 3:**
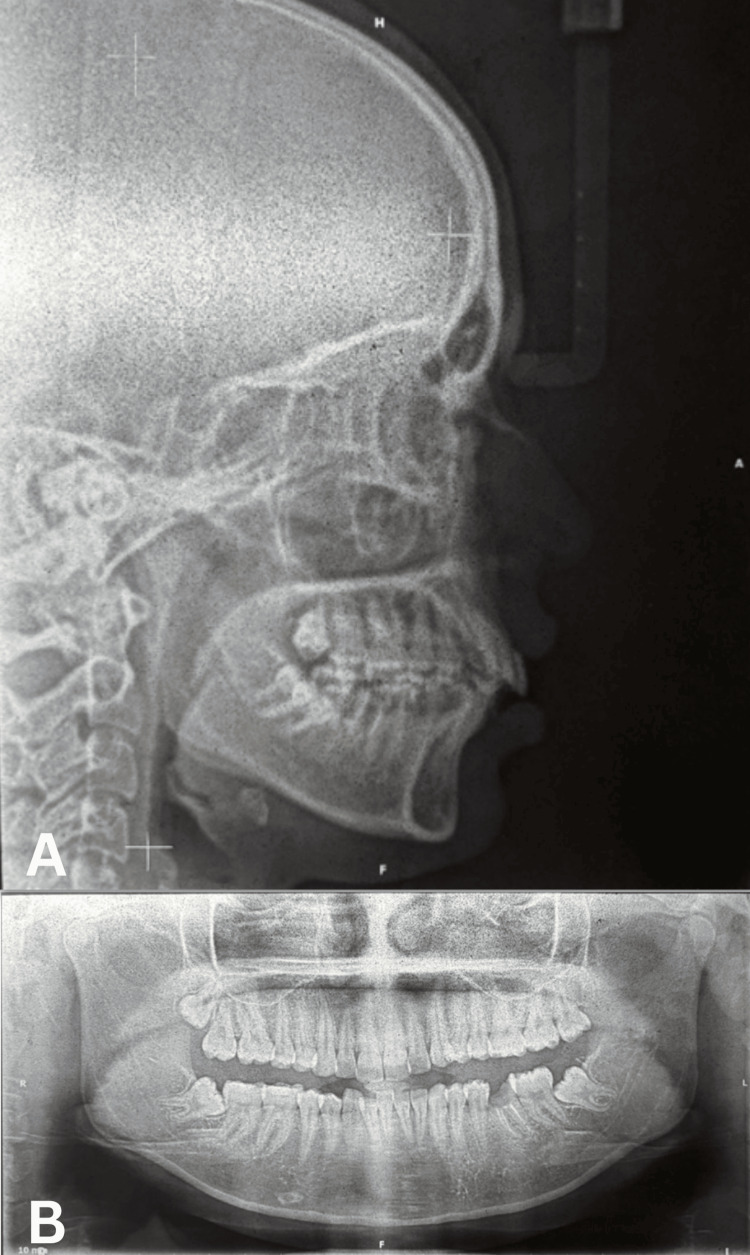
Pre-treatment radiographs: (A) Lateral cephalogram demonstrating vertical maxillary excess and proclined upper incisors. (B) Orthopantomogram showing decayed mandibular first molar (tooth 36) and mesioangularly erupting third molar (tooth 38).

Lateral cephalogram: Cephalometric analysis revealed VME with proclined upper incisors contributing to the gummy smile. The SNA angle measured 85°, SNB angle 79°, and ANB angle 6°, indicating a skeletal Class II maxillomandibular relationship. The Wits appraisal was +6 mm, confirming the sagittal discrepancy. Upper incisor to NA angle was 28° with 6 mm linear measurement, indicating proclination. The lower incisor to NB angle was 36° with an 8 mm linear measurement, showing significant proclination. The interincisal angle was reduced to 110°. Vertical parameters showed FMA of 24°, SN-GoGn of 30°, and Jarabak’s ratio of 66%, indicating a normal to slightly hypodivergent growth pattern. The occlusal plane cant measured at 5°. The Y-axis was increased at 67°, contributing to the VME (Figure 3a, Table 1).

OPG: The panoramic radiograph confirmed the presence of a severely decayed mandibular first molar (tooth 36) with extensive carious involvement extending to the pulp chamber, indicating a non-restorable status. The mandibular third molar (tooth 38) was visible in a mesioangular position. All other teeth were present with no significant pathology (Figure 3b, Table 1).

**Table 1:** Cephalometric analysis: pre-treatment and post-treatment comparison Comprehensive cephalometric measurements comparing pre-treatment and post-treatment values for sagittal, vertical, and additional cephalometric parameters. Standard/normal values are provided for reference. Key improvements include reduced ANB angle, improved incisor inclination, better vertical proportions, reduced occlusal plane cant, and enhanced facial balance.

Sr. no	Parameter	Standard/normal	Pre-treatment	Post-treatment
Sagittal relation				
1	SNA	82°	85°	85°
2	SNB	80°	79°	80°
3	ANB	2°	6°	+5°
4	WITS	0 to -1	6 mm	+12 mm
5	U1-NA	22°	28°	27°
6	U1-NA (mm)	4	6	2
7	L1-NB	25°	36°	29°
8	L1-NB (mm)	4	8	8
9	IMPA	90°	107°	98°
10	Interincisal angle	131°	110°	118°
Vertical				
1	FMA	25°	24°	34°
2	SN-Go Gn	32°	30°	30°
3	Jarabak’s ratio	62%-65%	66%	69%
4	Cant of occlusal plane	9°	5°	4°
5	Y-axis	59°	67°	63°
6	Lower lip to E-line	-2 mm	+3 mm	3 mm
7	Nasolabial angle	90°-110°	92°	124°
Additional cephalometric parameters				
	Vertical			
1	N perpendicular to A	0-2 mm	2 mm	1 mm
2	N perpendicular to Pog	0 to -6 mm	-7 mm	11 mm
3	Beta angle	27°-35°	25°	30°
4	Upper incisor to N perpendicular	4 mm	8 mm	2 mm
5	Lower incisor to A Pog	1 mm	5 mm	3 mm
6	Upper pharyngeal space	17 mm	12 mm	16 mm
7	Lower pharyngeal space	12 mm	6 mm	6 mm

IOPA: IOPA of the mandibular first molar confirmed the non-restorable dental status with extensive carious destruction and periapical involvement, necessitating extraction.

Diagnosis

Based on comprehensive clinical and radiographic evaluation, the patient was diagnosed with skeletal Class II maxillomandibular relationship (ANB 6°, Wits +6 mm), VME with excessive gingival display (>4 mm), occlusal cant on the right side (5°), deep bite (4 mm) with increased overjet (5 mm), Class II molar and canine relation on the left side, Class I relation on the right side, non-restorable mandibular first molar (tooth 36) requiring extraction, mesioangularly erupting mandibular third molar (tooth 38), dental midline discrepancy, proclined upper and lower incisors with flat smile arc and compromised smile esthetics.

Treatment

The objectives of the treatment were to reduce excessive gingival display and manage VME orthodontically without surgical intervention, correct occlusal cant and achieve smile symmetry, intrude anterior teeth and control vertical dimensions using skeletal anchorage, extract the decayed mandibular molar and manage space orthodontically, protract mandibular posterior teeth to avoid prosthetic replacement or implant placement, achieve ideal overjet (2-3 mm) and overbite (2-3 mm), coordinate dental midlines, establish bilateral Class I molar and canine occlusion, improve smile arc and facial esthetics, maintain periodontal health, and achieve stable occlusion.

Treatment plan

A comprehensive non-surgical orthodontic treatment plan was formulated with the extraction of the non-restorable mandibular first molar (tooth 36), protraction of mandibular second and third molars (teeth 37 and 38) into the extraction space using sliding mechanics with controlled anchorage, and bilateral extraction of maxillary premolars to facilitate whole arch intrusion and correct sagittal discrepancies. TADs were placed in the maxillary anterior region and IZCs in the maxillary posterior region to provide absolute anchorage. Maxillary anterior teeth were intruded using light continuous forces (15-20 g per tooth) with intrusion arches and wire sequencing [7-9]. Vertical dimensions were controlled through rectangular stainless steel wires and proper torque expression. Occlusal cant was corrected using asymmetric mechanics, controlled vertical elastics, and differential wire bends [13]. Comprehensive fixed appliance mechanotherapy, with proper bracket positioning and wire sequencing, was implemented to optimize smile esthetics, occlusal contacts, and root parallelism. Fixed and removable retention protocols were used for maintaining the outcome.

Treatment progress

Treatment was initiated with the extraction of the compromised mandibular first molar (tooth 36). Following extraction, a healing period of 4-6 weeks was allowed before initiating orthodontic space closure. Bonding of fixed orthodontic appliances (0.022-inch slot MBT prescription) was completed in both arches.

Initial Alignment Phase

Treatment began with 0.014-inch nickel-titanium (NiTi) archwires for initial alignment, followed by progression through 0.016-inch, 0.018-inch, and 0.016 × 0.022-inch NiTi wires to achieve proper alignment and leveling.

Molar Protraction Phase

Space closure was planned through strategic molar protraction using sliding mechanics. The mandibular second molar (tooth 37) and third molar (tooth 38) were protracted into the extraction space using 0.019 × 0.025-inch stainless steel archwires with controlled anchorage. Elastic chain and NiTi coil springs were used to generate appropriate forces (150-200 g) for bodily tooth movement. Careful monitoring ensured proper root parallelism and prevented undesirable tipping [10-12].

Maxillary Intrusion Phase

Following maxillary premolar extractions, TADs were placed in the maxillary anterior region between the lateral incisors and canines, and IZCs were placed in the infrazygomatic crest region bilaterally. Anterior intrusion mechanics were initiated using light continuous forces (15-20 g per tooth) delivered through intrusion arches connected to the TADs. Whole arch intrusion was facilitated by the IZCs in the posterior region, allowing simultaneous control of anterior and posterior vertical dimensions [7-9].

Occlusal Cant Correction

Occlusal cant correction was carried out using unilateral mechanics, controlled vertical elastics on the right side, and differential wire bends as needed. Asymmetric force application allowed gradual leveling of the occlusal plane and correction of the cant [13].

Finishing Phase

Rectangular stainless steel wires (0.019 × 0.025 inch) were used for torque control, root parallelism, and final detailing. Settling elastics were employed to optimize occlusal contacts and ensure proper intercuspation.

**Figure 4:**
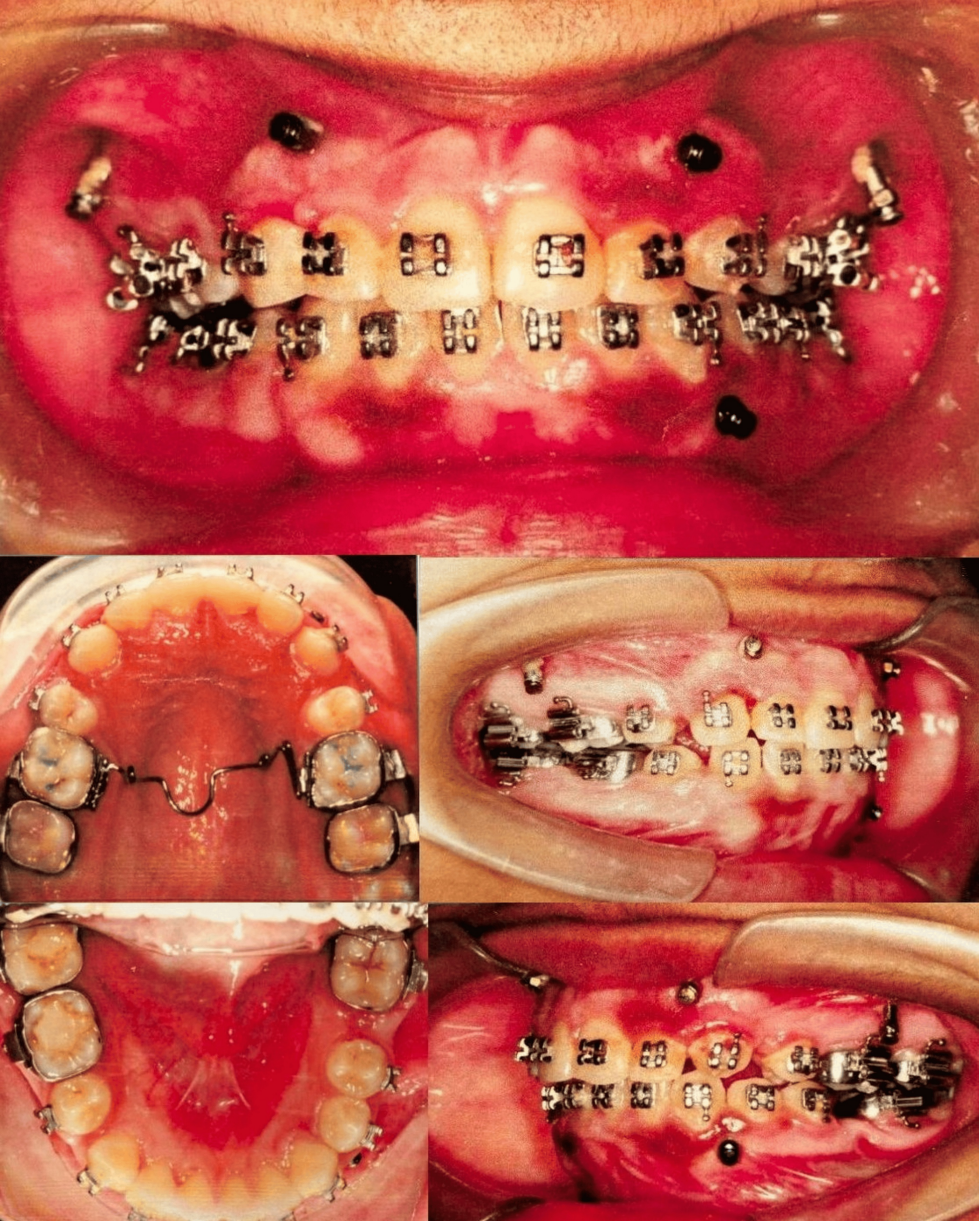
Mid-treatment intraoral photographs (progress photographs) showing orthodontic appliances in place, TADs and IZCs for skeletal anchorage, molar protraction in progress, and anterior intrusion mechanics.

Progress photographs demonstrated significant improvement with reduced gingival display, improved occlusal cant, successful protraction of the lower molar space without tipping, and a harmonized smile arc (Figure 4).

Treatment results

Post-treatment Extraoral Findings

**Figure 5:**
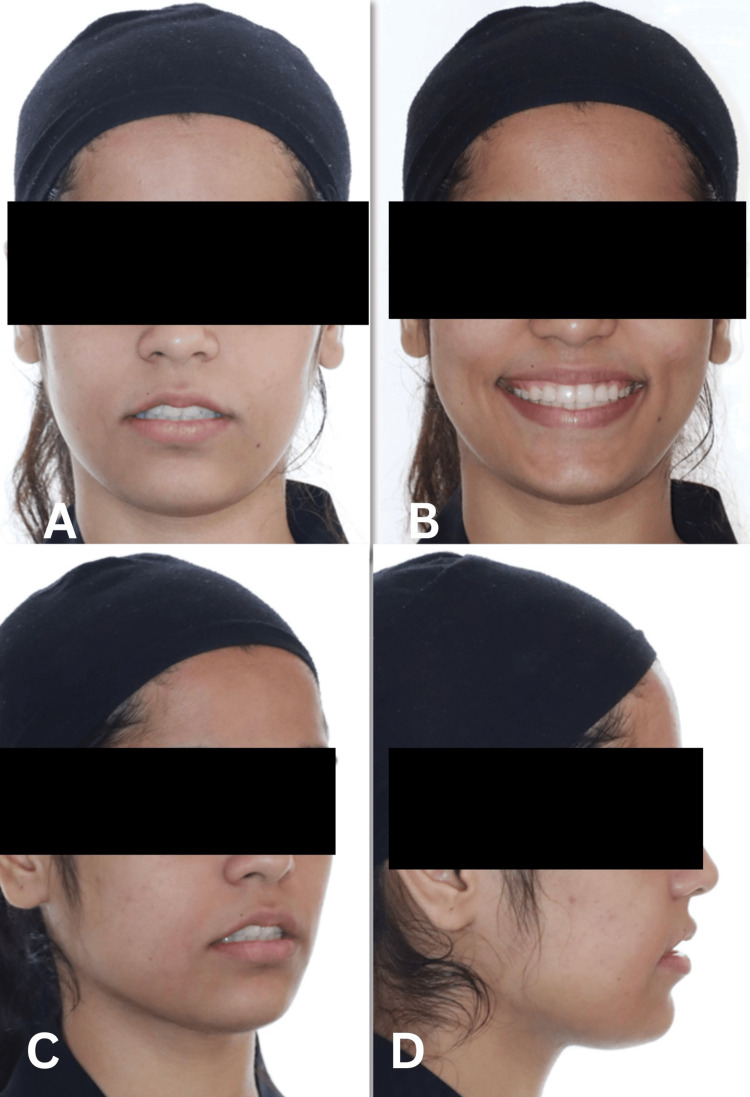
Post-treatment extraoral photographs (frontal and profile views) showing reduced gingival display (<2 mm), improved vertical facial balance, symmetrical smile arc, corrected occlusal cant, and enhanced dental and gingival esthetics.

Post-treatment facial evaluation showed significant improvement in smile esthetics and facial balance. Gingival display was reduced to less than 2 mm during smiling, achieving an esthetic smile line. Vertical facial balance improved with better proportions of the lower facial third. The smile width and arc became symmetrical, with correction of the occlusal cant. Dental and gingival esthetics were markedly enhanced, with improved incisor display and smile harmony (Figure 5).

Post-treatment Intraoral Findings

**Figure 6:**
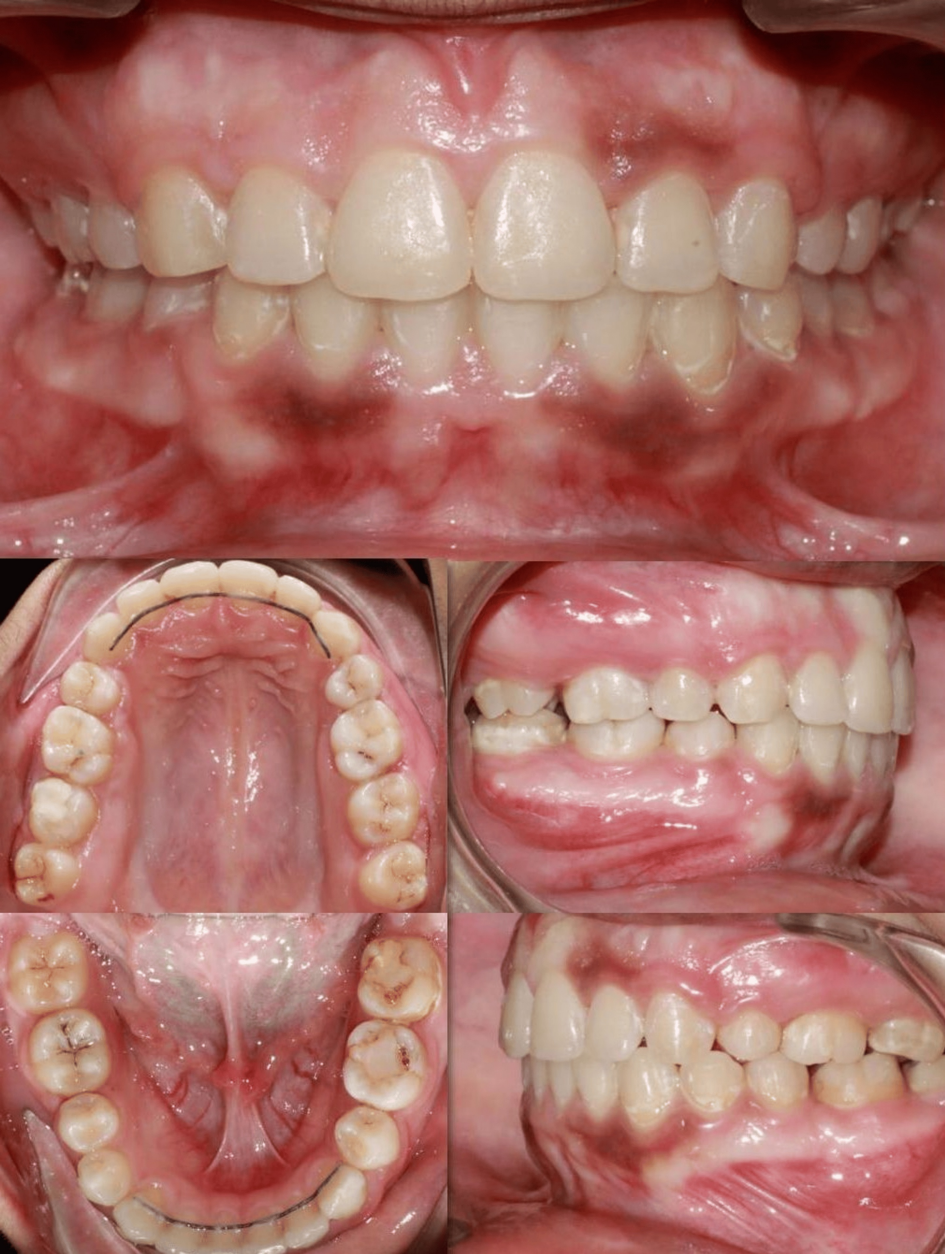
Post-treatment intraoral photographs (intraoral views) demonstrating bilateral Class I molar and canine relationships, complete space closure with successful molar protraction, ideal overjet (2 mm) and overbite (2 mm), coincident midlines, and stable occlusion.

Intraoral examination revealed successful mandibular molar protraction with complete space closure and no residual extraction space. Bilateral Class I molar and canine relationships were achieved. Overjet was corrected to 2 mm and overbite to 2 mm, both within ideal ranges. Dental midlines were coincident with the facial midline. The curve of Spee was flattened, and stable occlusion with proper intercuspation was established. Periodontal health was maintained throughout treatment (Figure 6).

Radiographic Evaluation

**Figure 7:**
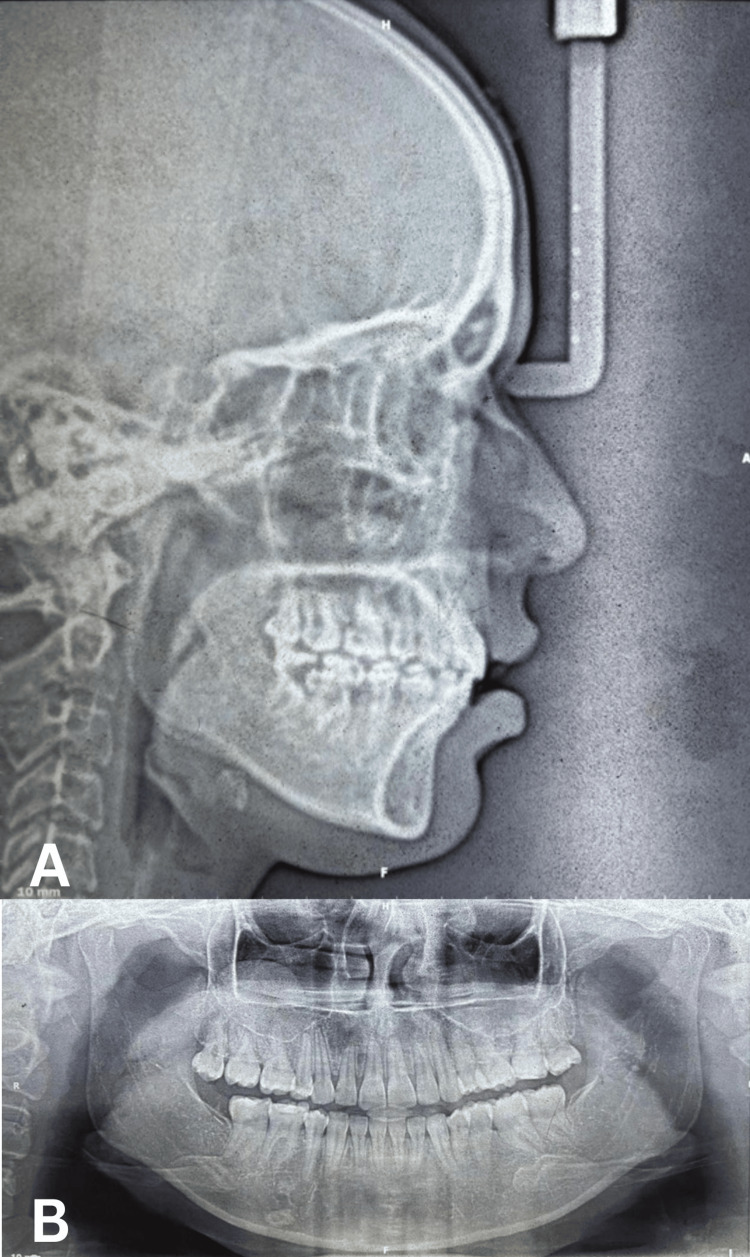
Post-treatment radiographs: (A) Lateral cephalogram showing controlled vertical dimension and improved incisor inclination. (B) Orthopantomogram confirming complete molar protraction without root resorption and proper root parallelism.

Post-treatment OPG: The panoramic radiograph confirmed complete protraction of the mandibular second and third molars into the extraction space without evidence of root resorption. Root parallelism was maintained, and bone levels were stable. All teeth demonstrated proper root angulation and healthy periodontal support (Figure 7b).

**Figure 8:**
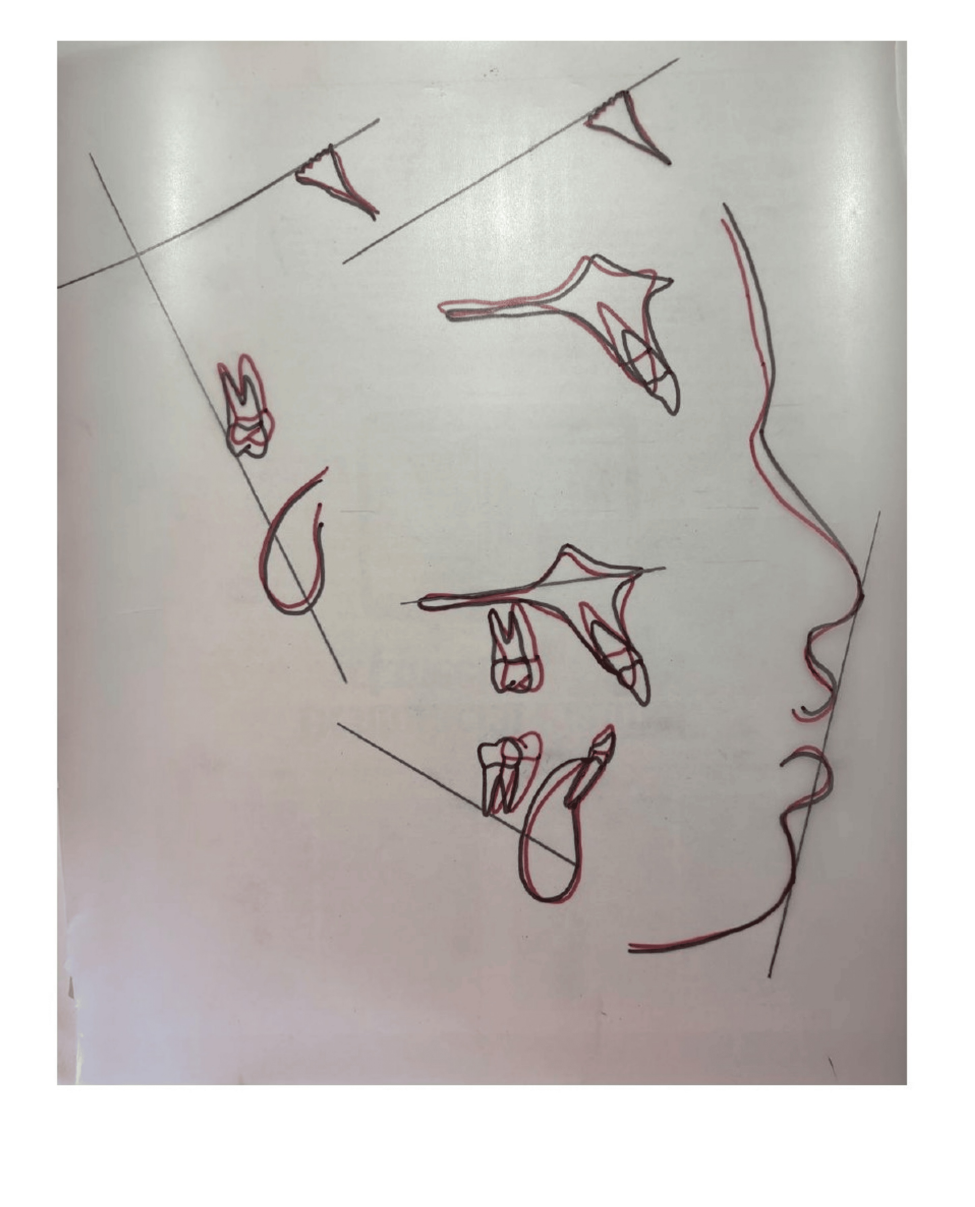
Cephalometric superimposition

Post-treatment lateral cephalogram: Cephalometric analysis showed controlled vertical dimension with reduction in gingival display. The SNA angle remained at 85°, SNB improved to 80°, and ANB reduced to 5°. The Wits appraisal increased to +12 mm due to mandibular autorotation. Upper incisor to NA angle was 27° with 2 mm linear measurement, indicating improved inclination. The lower incisor to NB angle was 29° with an 8 mm linear measurement. The interincisal angle improved to 118°. Vertical parameters showed FMA of 34°, SN-GoGn of 30°, and Jarabak’s ratio of 69%. The occlusal plane cant was reduced to 4°. The Y-axis improved to 63°, reflecting better vertical control. The nasolabial angle increased to 124°, indicating improved upper lip position (Figures 7a, 8 and Table 1).

Retention

A comprehensive retention protocol was implemented, including maxillary and mandibular fixed lingual retainers bonded from canine to canine, supplemented by removable clear retainers for nighttime wear. The patient was instructed on proper retainer care and scheduled for regular retention follow-up appointments.

## Discussion

Adult orthodontics poses numerous challenges due to mutilation in dentition, time constraints, and skeletal issues prevailing in malocclusion. It becomes prudent to address these challenges in an effective manner that best suits the estimated treatment goals. VME significantly affects esthetics, especially in young adults who are highly concerned with smile appearance. Excessive gingival display is not considered esthetic and can significantly impact self-esteem and quality of life.

Non-surgical management of VME

Orthodontic management of VME - although limited compared to orthognathic surgery - can produce substantial improvement when carefully planned [1-3]. The key highlight in this case is the dual management approach: (a) non-surgical vertical control using anterior intrusion and occlusal cant correction, and (b) molar protraction into an extraction space instead of opting for implant or prosthetic restoration [10-12].

Traditional treatment for severe VME typically involves orthognathic surgery, specifically Le Fort I maxillary impaction. However, orthognathic surgery is associated with inherent risks, including surgical complications, prolonged recovery, significant cost, and patient reluctance. Taking cognizance of the invasiveness of surgical procedures, a minimally invasive treatment plan was chosen after obtaining informed consent from the patient. The estimated tooth movements were initiated, taking into account the maximum possible permeability within the envelope of discrepancy [14].

Role of TADs

TADs and IZCs provide a new horizon of absolute anchorage, thereby avoiding taxation of any dental unit [4-6]. TADs in the anterior region and IZCs placed in the posterior region helped facilitate simultaneous whole arch intrusion, thus enabling the reduction of gummy smile. The use of skeletal anchorage allows for controlled intrusion mechanics with light continuous forces (15-20 g per tooth), which are critical for achieving true intrusion without extrusion of posterior teeth [7-9].

The success of TAD-assisted intrusion depends on proper placement, adequate primary stability, and appropriate force application. In this case, TADs were placed in the maxillary anterior region between the lateral incisors and canines, providing direct anchorage for anterior intrusion. IZCs were placed bilaterally in the infrazygomatic crest region, offering robust anchorage for whole arch intrusion and vertical control. This combination allowed for comprehensive vertical dimension management without compromising anchorage or causing undesirable side effects.

Molar protraction versus prosthetic replacement

The management of long-standing extraction cases presents a clinical dilemma between prosthetic replacement and molar protraction. It becomes imperative to oversee the time required for executing any treatment protocol while keeping cognizance of functional parameters and oral health-related quality of life. Molar protraction is a biologically favorable method that preserves bone, eliminates prosthetic needs, and maintains periodontal integrity [10-12].

In this case, the mandibular second molar (tooth 37) and third molar (tooth 38) were successfully protracted into the extraction space of the first molar (tooth 36). This approach offered several advantages: (1) preservation of natural dentition, (2) maintenance of alveolar bone volume, (3) elimination of prosthetic complications, (4) cost-effectiveness, and (5) long-term biological stability. The protraction was achieved using sliding mechanics with controlled anchorage, ensuring bodily tooth movement and proper root parallelism.

Alternative treatment options for the missing mandibular molar included: (1) implant-supported crown, (2) fixed partial denture, or (3) leaving the space open. Each option has limitations: implants require adequate bone volume and healing time, fixed partial dentures necessitate preparation of adjacent teeth, and leaving the space open can lead to drifting and occlusal instability. Orthodontic space closure through molar protraction emerged as the most biologically sound and functionally optimal solution.

Occlusal cant correction

Occlusal cant correction adds complexity to treatment planning, requiring asymmetric mechanics and controlled vertical elastics to achieve facial symmetry [13]. In this case, the occlusal cant was corrected using unilateral mechanics on the right side, where the cant was most pronounced. Differential wire bends and controlled vertical elastics allowed gradual leveling of the occlusal plane. The use of TADs and IZCs provided stable anchorage for asymmetric force application without causing undesirable tooth movements in other areas.

Alternative treatment approaches

Other options to reduce a gummy smile include botulinum toxin (Botox®) injections or orthognathic surgery [15]. Botulinum toxin injections into the levator labii superioris muscle can temporarily reduce the gingival display by limiting upper lip elevation. However, this approach is associated with increased risk of relapse, requires frequent follow-up visits (every 3-6 months), and does not address underlying skeletal or dental discrepancies. Orthognathic surgery, specifically Le Fort I maxillary impaction, provides definitive correction but is associated with surgical risks, prolonged recovery, and significant cost.

The orthodontic approach used in this case offered a minimally invasive alternative that addressed both the vertical excess and the underlying dental discrepancies. The patient showed marked improvement in smile line, gingival exposure, and overall harmony between teeth and soft tissue, demonstrating that carefully planned orthodontic mechanics can achieve significant esthetic improvement without surgical intervention.

Biomechanical considerations

Thorough planning and meticulous execution of planned treatment mechanics while taking utmost care of orthodontic biomechanics can help achieve a significant reduction of gummy smile in such borderline cases. Key biomechanical principles applied in this case included: 1. Light continuous forces: Intrusion forces of 15-20 g per tooth were maintained to achieve true intrusion without root resorption or anchorage loss [7-9]. 2. Absolute anchorage: TADs and IZCs provided skeletal anchorage, eliminating unwanted reciprocal tooth movements and allowing precise control of tooth position. 3. Controlled molar protraction: Forces of 150-200 g were applied for molar protraction using sliding mechanics, with careful monitoring to prevent tipping and ensure bodily movement [10-12]. 4. Asymmetric mechanics: Unilateral force application and differential wire bends allowed correction of the occlusal cant without affecting the contralateral side [13]. 5. Proper wire sequencing: Progressive wire sequencing from flexible NiTi to rigid stainless steel wires ensured proper alignment, leveling, and torque control throughout treatment.

Clinical implications and limitations

This case demonstrates that orthodontic management of VME-associated gummy smile, occlusal cant, and compromised molars can yield excellent esthetic and functional results without surgical intervention. However, certain limitations must be acknowledged: 1. Patient selection: This approach is most suitable for borderline cases with moderate VME. Severe skeletal discrepancies may still require orthognathic surgery for optimal results. 2. Treatment duration: Orthodontic space closure through molar protraction requires extended treatment time (24-30 months) compared with prosthetic replacement. 3. Patient compliance: Success depends on patient compliance with TAD/IZC maintenance, elastic wear, and retention protocols. 4. Skeletal anchorage: TAD and IZC placement requires adequate bone volume, proper surgical technique, and careful maintenance to prevent failure. 5. Stability: Long-term stability of intrusion results requires proper retention and may show some relapse over time.

Despite these limitations, the orthodontic approach offers significant advantages in terms of biological preservation, cost-effectiveness, and patient acceptance. The results achieved in this case demonstrate that carefully planned biomechanics and vertical smile management can address complex esthetic imbalances in adult orthodontic patients.

Future directions

Advances in skeletal anchorage systems, digital treatment planning, and biomechanical understanding continue to expand the possibilities for non-surgical management of vertical discrepancies. Three-dimensional imaging and virtual treatment planning allow for more precise prediction of treatment outcomes and optimization of TAD/IZC placement. Customized orthodontic appliances and individualized force systems may further enhance treatment efficiency and predictability.

## Conclusions

This case demonstrates that orthodontic management of VME-associated gummy smile, occlusal cant, and compromised molars can yield excellent esthetic and functional results without surgical intervention. Non-surgical VME management involves carefully planned orthodontic mechanics using TADs and IZCs, which can achieve a significant reduction in gingival display and improvement in smile esthetics without orthognathic surgery. Extraction followed by molar protraction is a viable, biologically conservative alternative to prosthetic replacement, preserving natural dentition and maintaining periodontal integrity. Skeletal anchorage systems allow effective gingival display reduction and smile improvement through controlled intrusion mechanics with light continuous forces. Successful management of complex adult orthodontic cases requires integration of multiple treatment modalities, including skeletal anchorage, differential intrusion, asymmetric mechanics, and strategic extractions. Minimally invasive orthodontic approaches offer acceptable alternatives to surgery for motivated patients with borderline skeletal discrepancies. This case highlights the importance of thorough diagnostic evaluation, comprehensive treatment planning, meticulous biomechanical execution, and patient-centered decision-making in achieving optimal esthetic and functional outcomes in adult orthodontic patients with VME and compromised dentition.
